# A structural decryption of cryptochromes

**DOI:** 10.3389/fchem.2024.1436322

**Published:** 2024-08-16

**Authors:** Cristina C. DeOliveira, Brian R. Crane

**Affiliations:** Department of Chemistry and Chemical Biology, Cornell University, Ithaca, NY, United States

**Keywords:** flavoprotein, light-sensing, photosensory receptor, signal transduction, circadian clock, redox chemistry, post-translational modification, protein oligomerization

## Abstract

Cryptochromes (CRYs), which are signaling proteins related to DNA photolyases, play pivotal roles in sensory responses throughout biology, including growth and development, metabolic regulation, circadian rhythm entrainment and geomagnetic field sensing. This review explores the evolutionary relationships and functional diversity of cryptochromes from the perspective of their molecular structures. In general, CRY biological activities derive from their core structural architecture, which is based on a Photolyase Homology Region (PHR) and a more variable and functionally specific Cryptochrome C-terminal Extension (CCE). The α/β and α-helical domains within the PHR bind FAD, modulate redox reactive residues, accommodate antenna cofactors, recognize small molecules and provide conformationally responsive interaction surfaces for a range of partners. CCEs add structural complexity and divergence, and in doing so, influence photoreceptor reactivity and tailor function. Primary and secondary pockets within the PHR bind myriad moieties and collaborate with the CCEs to tune recognition properties and propagate chemical changes to downstream partners. For some CRYs, changes in homo and hetero-oligomerization couple to light-induced conformational changes, for others, changes in posttranslational modifications couple to cascades of protein interactions with partners and effectors. The structural exploration of cryptochromes underscores how a broad family of signaling proteins with close relationship to light-dependent enzymes achieves a wide range of activities through conservation of key structural and chemical properties upon which function-specific features are elaborated.

## Introduction

**FIGURE 1:**
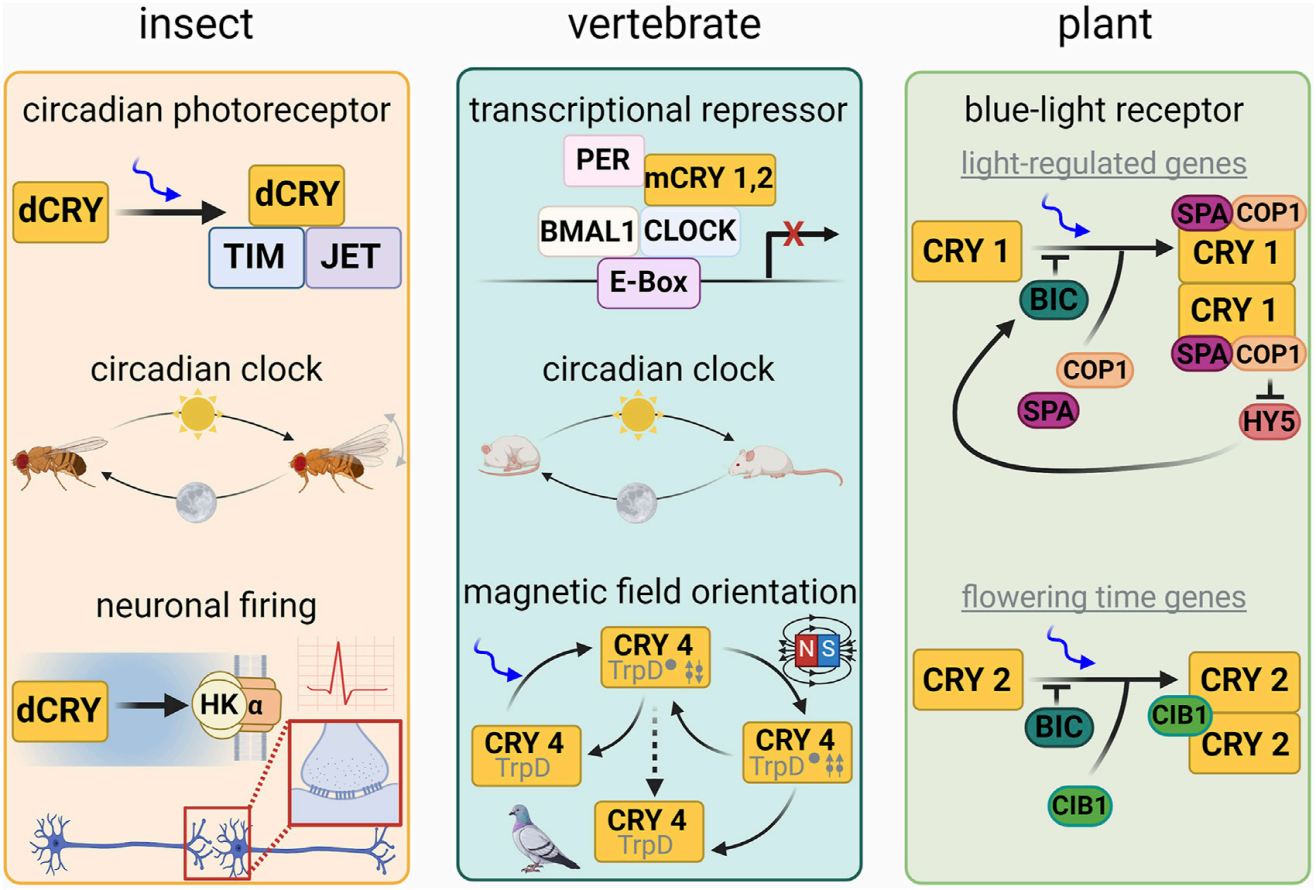
Cryptochrome function in different model organisms. In *Drosophila melanogaster*, dCRY acts as a circadian clock photoreceptor by coordinating light-dependent degradation of Timeless (TIM) by the E3 ubiquitin ligase Jetlag. dCRY also interacts with the potassium ion channel β-subunit Hyperkinetic to regulate neuronal firing. In vertebrates, cryptochromes acts as a transcriptional repressor and regulator of the circadian clock. In plants, CRY1 acts as a blue-light receptor in the regulation of light-regulated genes associated with growth and development and couples to circadian clock regulation, whereas CRY2 regulates genes governing flowering time. CRY has also been implicated in sensing earth’s magnetic field in invertebrates, plants, birds and certain mammals. Lower schematic of the central panel represents the proposed radical pair (twin arrows) mechanism involved in magnetoreception.

Cryptochromes represent a functionally diverse family of signal transduction proteins that are evolutionarily linked to light-dependent DNA repair enzymes known as photolyases (Sancar, 2003; Chaves, et al., 2011a; Zoltowski, et al., 2011a; Conrad, et al., 2014; Michael, et al., 2017a; Wang and Lin, 2020a; Foley and Emery, 2020; Kiontke, Stephan, et al., 2020). Light provides a ubiquitous environmental cue that shapes many biological processes, from circadian rhythms to developmental pathways (Zoltowski, et al., 2011b; Conrad, et al., 2014; Ahmad, 2016; Wang and Lin, 2020b). Many cryptochromes bind flavin cofactors and in doing so play a key role in light responses for many types of organisms (Figure 1). Other cryptochrome activities are light independent; hence, the utility of their structural framework goes beyond photobiology (Figure 1). Structures of diverse cryptochromes in complex with partner proteins and small molecules have revealed commonalities and differences in their modes of recognition and ability to propagate signals to their targets (Conrad, et al., 2014; Wang and Lin, 2020a; Foley and Emery, 2020). Herein we examine the structural biology of cryptochromes to uncover broad insights into the fundamental mechanisms that underlie the modulation of dynamic protein interactions by reactive cofactors.

## Evolutionary relationships of cryptochromes: signaling proteins and photoenzymes

**FIGURE 2:**
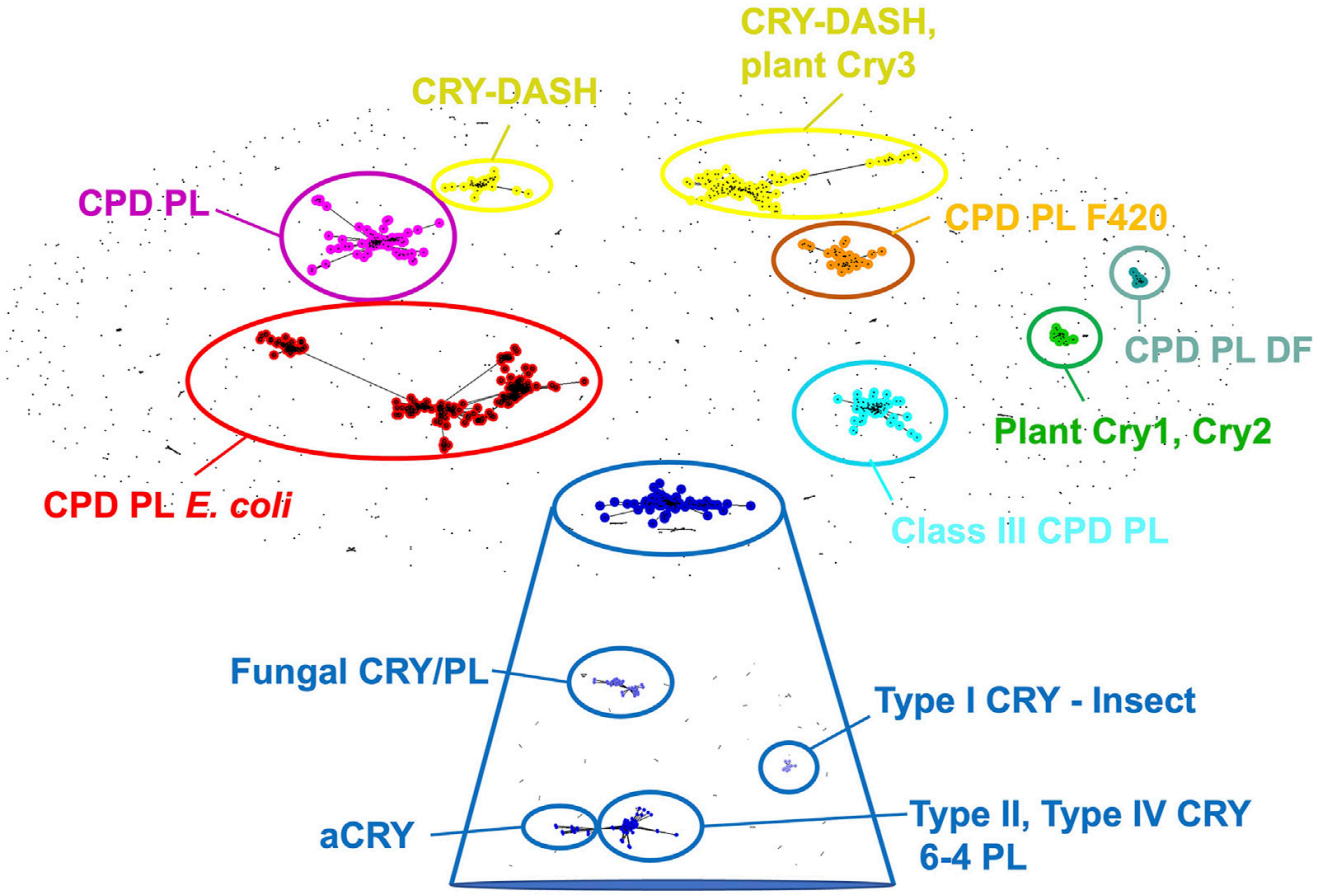
Cryptochrome and photolyase sequence clusters. When clustered by pair-wise sequence similarity, cryptochromes are sorted into CPD photolyases, CPD photolyases as found in *E. coli*, CPD photolyases containing the F420 cofactor, CPD photolyases DF, Class III CPD photolyases, CRY-DASH, CRY-DASH and plant CRY3, plant CRY one and 2, and a CRY cluster that upon more stringent differentiation (expanded blue box) segregates into categories represented by fungal CRYs, animal-like algal CRYs (aCRYs), Type 1 insect CRYs (e.g., dCRY), as well as vertebrate Type II, Type IV CRYs and 6-4 photolyases. Sequence clustering performed by clans (Frickey and Lupas, 2004).

Originally discovered in plants and flies, cryptochromes exhibit considerable sequence similarity with DNA photolyases (PLs) (Aguida, et al., 2024; Ahmad and Cashmore, 1993; Balland, et al., 2009; Emery, et al., 1998; Kanai, et al., 1997; Ozturk, N., et al., 2007; Sancar, 2003; Stanewsky, et al., 1998). Photolyases repair cross-linked pyrimidine dimers in UV-damaged DNA using light-dependent redox reactions mediated by their flavin cofactors (Carell, et al., 2001; Essen and Klar, 2006; Sancar, 2003). Depending on the identity of pyrimidine base lesions that they recognize photolyases fall into two functional types: cyclic pyrimidine photolyases (CPDs) and 6-4 photolyases (Carell, et al., 2001; Sancar, 2003; Essen and Klar, 2006). Although cryptochromes have evolutionarily diversified into a wide array of roles that extend beyond these activities, including circadian regulation and phototaxis, sequence homology to photolyases remains high (Chaves, et al., 2011b; Essen, et al., 2017; Ozturk, N., 2017). Moreover, photolyase and cryptochrome sequences across all kingdoms of life do not segregate into clear functional groups, emphasizing the close relationships within the greater family (Chaves, et al., 2011a; Ozturk, N., 2017) (Figure 2). Sequence clustering of the CRY/PL family reveals distinct groups of both proteins, such as the Class I and Class II CPD photolyases, distinguished by their antenna cofactors, and the so-called Class III photolyases (Figure 2) that are more closely related to plant cryptochromes (Kiontke, S., et al., 2014; Ozturk, N., 2017). DASH-CRYs (**D**rosophila, **A**rabidopsis, **S**ynechocystis, **H**uman-type cryptochromes), which are found in bacteria, algae or associated with organelles of higher plants (Brudler, et al., 2003; Kleine, et al., 2003; Essen, et al., 2017; Kiontke, Stephan, et al., 2020) may possess both signaling and DNA repair activities. DASH-CRYs segregate into two closely related groups (Figure 2): one that includes the plastid CRY3 from *Arabidopsis thalania* (Kleine, et al., 2003; Klar, et al., 2007) and other bacterial CRY-DASH proteins. In the major sequence category of cryptochromes, which also contains the 6-4 photolyases, delineations emerge among the Type I invertebrate CRYs, Type II and Type IV vertebrate CRYs under stricter clustering criteria (Figure 2), whereas land plant CRYs (Wang and Lin, 2020a), as represented by *A. thalania* CRY1 (AtCRY1) and CRY2 (AtCRY2), stand apart, owing in part to their extensive CCEs.

## Types of cryptochromes: from circadian rhythms to magnetic field sensing

Cryptochromes exhibit a wide range of functions, which is in contrast to relatively modest diversification of their structural and enzymatic features (Figure 1). Type I invertebrate CRYs entrain circadian rhythms to light by interacting with core elements of the circadian oscillator. Type II CRYs, including those from mammals, are unlikely to be light sensors, bind FAD weakly and may not require the cofactor for their functional roles (Sancar, 2004; Kutta, et al., 2017; Calloni and Vabulas, 2023). Recent work however does suggest that mammalian CRYs (mCRYs) may bind flavin as a mechanism to regulate and protect against ubiquitin-mediated degradation (Hirano, et al., 2017) and interestingly human CRYs (hCRYS) can rescue some presumably light-dependent functions of *Drosophila melanogaster* CRY (dCRY) in transgenic fly lines (summarized in (Vanderstraeten, et al., 2020)). Type II CRYs are also key components of the circadian clock, but rather than act in light entrainment, they compose the repressor complexes that feedback to inhibit the positive-acting heterodimeric transcriptional activators of the clock (CLOCK:BMAL1) (Sancar, 2004; Partch, Carrie L., et al., 2014; Michael, et al., 2017a; Michael, et al., 2017b; Takahashi, 2017). (Note that the abbreviation “mCRY” is often used to refer to “mouse” CRY, with the mouse being a key experimental system to study circadian rhythms; here we use the more general definition of mammalian CRY (mCRY), and further designate non-mouse mCRYs where appropriate). Type IV CRYs (Wang, et al., 2018a), found in reptiles, amphibians, fish, and birds, bind FAD and may participate in the sensing of the earth’s magnetic field for navigation during migratory behavior (Xu, J. J., et al., 2021), as do Type I CRYs in invertebrates (Gegear, et al., 2008; Maeda, et al., 2008; Hiscock, et al., 2016; Agliassa, et al., 2018; Zoltowski, et al., 2019). Land plant CRY1 and CRY2 (e.g., AtCRY1 and AtCRY2 from *Arabidopsis*) are associated with growth, development, flowering, circadian rhythms and magnetic field sensitivity in plants (for reviews see (Galland and Pazur, 2005; Thoradit, et al., 2023)). Non-circadian-clock-related functions of Type I cryptochromes include the modulation of neuronal firing frequency, UV light avoidance in insects, and sensing of the lunar cycle (Baik, et al., 2019; Baik, et al., 2017; Damulewicz and Mazotta, 2020; Fogle, K. J., et al., 2015; Fogle, K.J., et al., 2011; Poehn, et al., 2022; Zurl, et al., 2022). Type II CRYs also play roles distinct from their repressive functions in circadian rhythms such as regulating the glucocorticoid receptor, cAMP signaling associated with gluconeogenesis (Lamia, et al., 2011; Tan and Scott, 2014; Zhang, E. E., et al., 2010) and the differentiation of brown adipose tissue (Miller, et al., 2020a; Miller, et al., 2021). Many organisms contain both Type I and Type II CRYs, whereas some contain only one or the other (Kotwica-Rolinska, et al., 2022). Notably the loss of Type I CRYs is usually associated with the loss of its target in the clock oscillator: the co-repressor Timeless (TIM) (Kotwica-Rolinska, et al., 2022). Animal-type CRYs (aCRYs) from algal species, act both as circadian clock regulators and 6-4 photolyases (Coesel, et al., 2009; Heijde, et al., 2010; Fortunato, et al., 2015; Essen, et al., 2017; Kottke, et al., 2017). The CRY-DASH family (or class 0 PLs) are found throughout archaea, bacteria, plants and even vertebrates, but not mammals, and have quite diverse functions in signaling and transcriptional regulation (Brudler, et al., 2003; Kleine, et al., 2003; Kiontke, Stephan, et al., 2020), maintaining DNA repair activity in some cases, particularly for single-stranded DNA (Selby and Sancar, 2006; Tagua, et al., 2015; von Zadow, et al., 2016).

Underscoring their commonalities, CRYs and PLs often have evolutionarily conserved functions, demonstrated by ectopic and *in vitro* experiments. The *Ostereococcus tauri* (algal) CRY-DASH will inhibit CLOCK:BMAL1-mediated activation of a circadian clock reporter gene (Heijde, et al., 2010). Portorous tridactylus CPD PL interacts with CLOCK and can restore transcriptional oscillations in the liver of clock-deficient *cry1*/*cry2* mice (Chaves, et al., 2011b). At high concentrations, a truncated form of AtCRY1 can repair the CPD lesion (Zwang, et al., 2018).

Clock-associated CRYs from various vertebrate species, link to functions such as nonvisual photoreception, solar compass orientation, and time–place learning, thereby highlighting the adaptability and versatility of cryptochrome function (Emery et al., 1998; Cermakian, et al., 2002; Cermakian et al., 2002; Van der Zee, et al., 2008; Van der Zee et al., 2008; Hitomi et al., 2009). Understanding the interplay between these different phenotypes enriches our comprehension of the broader ecological roles and evolutionary significance of cryptochromes.

## Cryptochrome structure: a base to build diverse functionality

**FIGURE 3:**
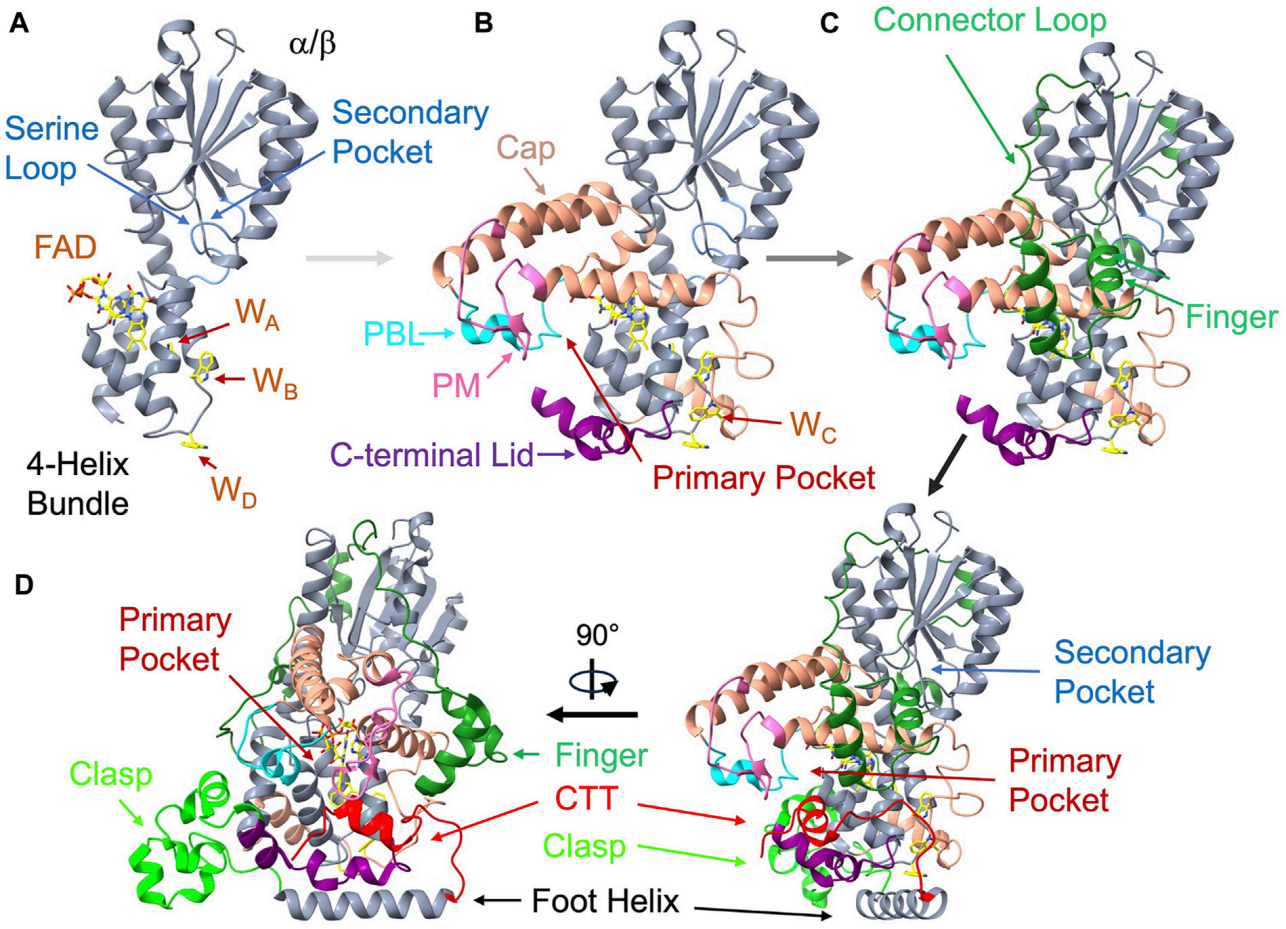
Structural anatomy of cryptochrome. The *Drosophila* CRY (dCRY) structure (PDB: 4GU5) assembled from its components. **(A)** On the left top the primase related 4-helix bundle (360-423, grey) binds the isoalloxazine ring below the α/β Rossman fold domain (residues 135-169, grey). The serine loop (light blue, 42-53) borders the secondary pocket. Three of the four redox active Trp residues (W_A_, W_B,_ W_D_) locate to the primase bundle. **(B)** Addition of the helical cap (salmon, 226-359) provides interactions to the adenosine moiety and harbors the phosphate-binding loop (PBL, cyan, 288-306) and the protrusion motif (PM, pink, 288-306). W_C_ is on a loop connecting to the bundle. The C-terminal lid (purple, 420-466) completes the primary pocket. **(C)** On the top right, the connector loop (dark green, 141-225), which contains the finger motif (dark green, 140-170) reaches across the α/β domain to connect the α-helical domain (green). **(D)** To complete the structure (lower right and rotated lower left), the clasp region (lime green, 470-496) follows the C-terminal lid, leads into the foot or CC helix (497-518), and finally the C-terminal tail (CTT, red, 519-542).

CRYs share an architecture with PLs known as the Photolyase Homology Region (PHR) that comprises two domains: the α/β domain and the α-helical domain (Park, et al., 1995; Brudler, et al., 2003; Brautigam, et al., 2004; Hitomi, et al., 2009). Below we describe how distinct elements compose the cryptochrome fold, referencing to the dCRY structure (Zoltowski et al., 2011a; Czarna, et al., 2013; Levy, et al., 2013) that has been decomposed into its structural elements in Figure 3 (note that the residue and secondary structure numbering may differ slightly for any given CRY, largely due to variability in loop regions). The α/β domain consists of a typical Rossman fold, where the traditional nucleotide diphosphate binding motif sits at the middle of the parallel β-sheet edge. The nucleotide binding region forms a cavity with the α-domain, wherein antenna cofactors, such as 5,10-methenyltetrahydofolate (MTHF), bind in PLs. In CRYs, this cavity forms the so-called secondary pocket that mediates interactions with partners, particularly the CLOCK:BMAL1 transcriptional activators in the circadian oscillators of mammals (Partch et al., 2014; Rosensweig, et al., 2018; Fribourgh, et al., 2020). A flexible loop that connects the second β-strand of the α/β domain to the second α-helix, known as the “serine loop” (residues 38–48 mCRY1, 42–53 dCRY), borders the secondary pocket and its dynamics modulate interactions between the mCRYs and CLOCK:BMAL1 (Fribourgh, et al., 2020).

Following the α/β domain a helix-turn-helix region (α5-α6, residues 135-169 in dCRY) curls like two fingers down against α11 of the α-helical domain before returning as a long connector to loop (170-266) that tracks back over the top of the entire α/β domain and then into the first helix, α8, of the α-helical domain. α8, α10 and α11 partially align to cap the top of the flavin cofactor by interacting with the adenosine moiety and FAD diphosphate groups and forming the primary pocket. Within this cofactor-capping region reside two loop elements that diverge in sequence and conformation among CRY homologs but have important functional and ligand binding properties: the phosphate binding loop (PBL, residues 249-263) following α8 and the protrusion motif (PM, residues 288-306) following α10 (Hitomi, et al., 2009). At the center of the α-helical domain, a skewed 4-helix bundle (α13-α16; residues 360-423) binds the isoalloxazine ring of the flavin adenine dinucleotide (FAD) cofactor at the center of its C-terminal three helices. CRYs and PLs share this core 4-helix bundle with DNA primase enzymes and hence these proteins may be related through an ancient connection involving single-stranded polynucleotide binding of primases and DNA repair activity of PLs and CRY-DASHs (Sauguet, et al., 2010).

Following the last helix of the bundle (α16), the Ser-rich (and in some cases, Cys-rich) extended C-terminal lid (residues 420-446) forms a cavity with the PBL and PM adjacent to the flavin. In dCRY this cavity accommodates the C-terminal tail (CTT) helix. The C-terminal lid, also known as the lid loop in mCRYs (residues 405-412 in mCRY1, residues 423-430 in mCRY2), partly determines specificity of the mCRY flavin pocket for targeted small molecules by influencing the conformations of conserved residues in the binding pocket (Miller, et al., 2020a; Miller, et al., 2020b; Miller, et al., 2021). In crystal structures, the lid-loop conformation is often ill-defined or influenced by crystal contacts. The C-terminal lid also contains key cysteine residues (mCRY1 Cys412 and Cys414) that play a role in regulating potentially redox-dependent interactions with the Period protein (PER) in mCRYs (Nangle, S. N., et al., 2014; Schmalen, et al., 2014). The C-terminal lid then leads into an irregular helical region that includes α18 and α19 (residues 449-497) that we will call the “clasp”. The clasp associates adjacent subunits in the higher oligomeric states of land plants. Finally, C-terminal to the clasp is a long foot helix (α20) that is the last conserved region of the PHR. For mCRYs, the foot helix was identified as a potential coiled-coil (CC) motif by sequence analysis (the CC-helix) (Chaves, et al., 2006). Appended to α20, the cryptochrome C-terminal extension (CCE) varies substantially among CRY homologs.

CRYs and PLs typically bind FAD (Lin, C. T., et al., 1995) in an unusual “U” shaped conformation that associates the adenosine with the isoalloxazine ring. FAD acts as the primary blue-light sensitive chromophore (Sancar, 2003; Berndt, et al., 2007; Chaves, et al., 2011a; Zoltowski, et al., 2011b) and the secondary pocket may bind antenna chromophores in some cryptochrome types to improve photosensitivity, likely for DNA repair activity (Kiontke, S., et al., 2014). The electrocatalytic reactions of PLs benefit from the U-shaped conformation of FAD that allows electronic communication between the adenosine and isoalloxazine moiety (Zhang, M., et al., 2017).

Most CRYs and PLs undergo photoreduction of their flavin cofactors when the singlet excited states of the flavin oxidize an adjacent Trp triad or tetrad of residues (Banerjee, et al., 2007; Biskup, et al., 2013; Giovani, et al., 2003; Kottke, et al., 2006; Kutta, et al., 2018; Lin, C. F., et al., 2018; Nohr, et al., 2016; Paulus, et al., 2015; Zeugner, et al., 2005). These residues propagate an electron hole to the surface of the protein by successive oxidation where it eventually reacts with external reductants to stabilize the reduced flavin. Three of these four Trp residues (A, B, C) reside in the primase 4-helix bundle, with A and B at the peripheral end of the last two helices, respectively, the third (C) situated on a connecting loop leading from α11 in the cofactor cap across the base of the bundle. Often CRYs conserve a fourth, even more solvent-exposed Trp residue (D) that acts as the terminal position of oxidation and resides on the loop connecting to the last two bundle helices (Biskup, et al., 2013; Kutta, et al., 2018; Lin, C. F., et al., 2018; Nohr, et al., 2016; Oldemeyer, et al., 2016; Paulus, et al., 2015). Alternations of this terminal Trp can completely abrogate stable flavoreduction and downstream partner engagement in dCRY (Lin, C., et al., 2022; Lin, C. F., et al., 2018; Nohr, et al., 2016). In addition, variations in the Trp tetrad are found across the PL/CRY family, particularly in proteobacterial CRYs (Geisselbrecht, et al., 2012) and Class II photolyases (Kiontke, S., et al., 2011). In some cases, the terminal Trp residues can be further augmented by an additional Tyr residue (Oldemeyer, et al., 2016; Zoltowski, et al., 2019; Vu, et al., 2023) and in others, alternative Trp-triads are operative (Kiontke, S., et al., 2011). In general, efficient photoreduction of the flavin requires at least a Trp triad (Kao, et al., 2008; Kiontke, S., et al., 2011; Lin, C. F., et al., 2018; Nohr, et al., 2016; Oldemeyer, et al., 2016). Notably, the initial charge separation reaction that localizes radical states on both the flavin and a tryptophan residue provides a basis for the radical-pair mechanism of magnetic field sensing proposed for avian migration and other CRY-mediated geomagnetic field responses (Ritz, et al., 2000; Rodgers and Hore, 2009; Hiscock, et al., 2016). Trp radicals in the Trp-tetrad may also have structural effects if they are sufficiently long-lived (Ma, et al., 2020a; Cellini, et al., 2024), although whether such changes have the ability to modulate partner interactions important for signal transduction remains to be determined.

Single-molecule folding studies on dCRY find tight coupling between FAD binding and polypeptide folding, with FAD interacting with largely unfolded intermediates at faster than diffusion-controlled rates and at high affinity (Foroutannejad, et al., 2023). Interactions of the isoalloxazine dominate this process, thereby indicating that the cofactor plays a role in structuring the primase-related 4-helix bundle, and perhaps capping region. Association of the CCE with the PHR appears to be the final folding step, in keeping with a low barrier to the dissociation of this element (Vaidya, et al., 2013; Berntsson, et al., 2019; Chandrasekaran, et al., 2021), which varies across CRY paralogs and described further below.

## CRY C-Terminal extensions (CCEs): structural versatility and functional impact

Positioned at the C-terminal end of the PHR, the CCE, (sometimes abbreviated as CCT) and also called a carboxyl-terminal extension (CTE), distinguishes CRYs from their PL counterparts (Chaves, et al., 2011b). CCEs vary in length (from ∼20 residues in invertebrate CRYs to ∼200 residues in plant CRYs) and often contain flexible regions that lack a well-defined, known tertiary structure (Partch, C. L., et al., 2005). These extensions exhibit structural plasticity, which allows them to modulate the interactions of the PHR, particularly the flavin pocket and serve themselves as a hub for protein-protein interactions (Chandrasekaran, et al., 2021; Chaves, et al., 2011a; Parico, et al., 2020; Partch, C. L., et al., 2005). Light often modulates conformation of the CCE and interactions of the CCE with the PHR.

In Type I invertebrate CRYs, such dCRY, a 23-residue helical CCE or CTT containing an 11-residue helix inserts into the flavin-binding pocket in the dark state. Interactions of the CTT with the flavin pocket prevent dCRY from engaging signaling partners until light undocks it (see below) (Berntsson, et al., 2019; Chandrasekaran, et al., 2021; Lin, C. F., et al., 2018; Vaidya, et al., 2013). Removal of the CTT generally activates dCRY (Busza, et al., 2004; Ceriani, et al., 1999; Dissel, et al., 2004; Hemsley, et al., 2007; Lin, C., et al., 2022).

The CCEs of mammalian CRYs also play key functional roles, although these mechanisms are not necessarily linked to flavin chemistry. With respect to maintaining circadian rhythms in mouse embryonic fibroblasts (MEFs), the CCEs of neither mCRY1 nor mCRY2 are required, yet they have substantial and differential effects on rhythm period length and amplitude (Khan, et al., 2012). Furthermore, sequencing of human subjects with delayed sleep phase disorder (DSPD) identified a genetic polymorphism that excised exon11 of mCRY1 (Patke, et al., 2017; Parico, et al., 2020). Loss of the exon 11 coding sequence shortens the CCE and increases interactions with and thereby repression of the transcriptional activators CLOCK:BMAL1 (Patke, et al., 2017). Biophysical experiments demonstrate that the segment coded by exon11 indeed interacts with the PHR and thereby blocks interaction with CLOCK (Parico, et al., 2020). Contacts between the PHR and the CCE are conserved in mCRY2 and modulate interactions with the mCRY2 binding partner mPER2 (Ozber, et al., 2010; Parico, et al., 2020). The specificity of small molecule inhibitors that have been targeted to the flavin pocket of mCRYs also depends on the CCE (Miller, et al., 2020a), suggesting interactions between the CCE and the flavin pocket where the molecules bind. Furthermore, phosphorylation of the mCRY1 CCE is linked to period length, likely because, as with plant CRYs (see below), phosphorylation affects its conformation and hence access to the primary pocket, where the E3 ubiquitin ligase FBXL3 targets mCRY (Gao, Peng, et al., 2013; Xing, et al., 2013). In addition to harboring a nuclear import signal (Chaves, et al., 2006), the mCRY1 CCE also makes important contacts in the transcriptional repression complex with CLOCK:BMAL1, competing with the BMAL1 transactivation domain (TAD) for binding coactivators (Chaves, et al., 2006; Xu, Haiyan, et al., 2015).

In another related example, aCRYs from algae (e.g., *Chlamydomonas reinhardtti*) contain a ∼100 residue CCE whose conformation gates interaction with the clock component Rhythm of Chloroplast (ROC) (Li, P., et al., 2022). aCRYs may function both in signaling and single-strand DNA repair (like some CRY-DASH proteins) (Essen, et al., 2017; Kottke, et al., 2017). The aCRYs are both blue and red-light sensitive because blue light drives the FAD to the neutral semiquinone, a state that then absorbs red light to be further photoreduced to the hydroquinone (Lacombat, et al., 2019; Li, P., et al., 2022; Oldemeyer, et al., 2016; Spexard, et al., 2014).

The avian CRYs associated with light-dependent magnetic field sensing (Niessner, et al., 2011; Xu, J. J., et al., 2021) also undergo conformational changes at their C-termini in response to light (Watari, et al., 2012; Niessner, et al., 2013; Niessner, et al., 2014; Zoltowski, et al., 2019). Avian CRY4 of chickens and CRY1a of both chickens and European robins localize to the retina (Niessner, et al., 2011; Watari, et al., 2012). A CRY1a epitope-specific antibody that targets the C-terminus only reacts with the protein in the discs of the retinal outer segments after illumination (Niessner, et al., 2013; Niessner, et al., 2014) and a C-terminally-directed chicken CRY4 antibody also reacts in a light-dependent manner (Watari, et al., 2012), thereby suggesting that the C-terminus becomes more accessible upon flavin photoreduction in both cases. However, biochemical studies of Type IV pigeon CRY4 (Zoltowski, et al., 2019) indicate rather that the C-terminus becomes more sequestered in light (see below).

Land plant cryptochromes (e.g., AtCRY1 and AtCRY2) have extensive and notably disordered CCEs (Partch, C. L., et al., 2005; Wang and Lin, 2020b). A wide variety of interacting proteins bind to AtCRY1 and AtCRY in both dark and light (see Table 1 in (Wang and Lin, 2020a; Qu, et al., 2024)). The CCEs themselves bind key targets (Yang, et al., 2000; Wang and Lin, 2020b): for example, the CCEs of AtCRY1 and AtCRY2, which differ considerably in sequence, both interact with the WD repeats of the ubiquitin ligases SPA/COP1, although the PHR domain of AtCRY2 also interacts with the kinase-like domain of COP1 (Lian, et al., 2011; Liu, B., et al., 2011; Liu, B. B., et al., 2016; Yu, et al., 2007; Zuo, et al., 2011). Light is proposed to release the CCEs from interaction with the PHRs so that both components can engage partners (Goett-Zink, et al., 2021; Kondoh, et al., 2011; Partch, C. L., et al., 2005; Yu, et al., 2007). It has long been recognized that the CCEs themselves exhibit signaling properties and when expressed alone confer constitutive activation of signaling pathways (Yang, et al., 2000; Yu, et al., 2007). A particular 80-residue region proximal to the PHR, called NC80, confers CRY2 functionality (Yu, et al., 2007). This motif is sequestered in the dark, but light-induced phosphorylation of the CCE exposes it for signal transduction (Yu, et al., 2007). The intrinsic disorder of the CCEs is thought to contribute to their signaling capabilities, allowing induced folding upon binding either by their targets or the cognate PHR domains (Michael, et al., 2017b; Partch, C. L., et al., 2005). Furthermore, the CCEs play a critical role in the stability of photobodies, which are a highly aggregated form of plant CRY (Más, et al., 2000; Wang, Q., et al., 2016; Yu, et al., 2009) with properties of liquid-liquid phase separation (LLPS) (Liu, Q. W., et al., 2024; Qu, et al., 2024). LLPS is primarily driven by the PHR domains, but light gating, and phosphorylation-associated stability of the condensates depend on the CCEs (Gao, L., et al., 2022; Liu, S. Y., et al., 2022; Ma, et al., 2023; Wang, et al., 2021a). These phase-separated states recruit and sequester a large number of protein partners (for an extensive summary see (Qu, et al., 2024)) and are considered to mediate an alternative mode of molecular recognition, distinct from those interactions mediated by complimentary 3-dimensional structure (Qu, et al., 2024). For example, AtCRY2 interacts with and regulates different classes of N^6^-methyladenosine (m6A) RNA methyltransferases via LLPS to influence m6A modifications and RNA stability (Jiang, et al., 2023; Wang,et al., 2021b).

## Primary and secondary pockets: interactions and signaling

**FIGURE 4:**
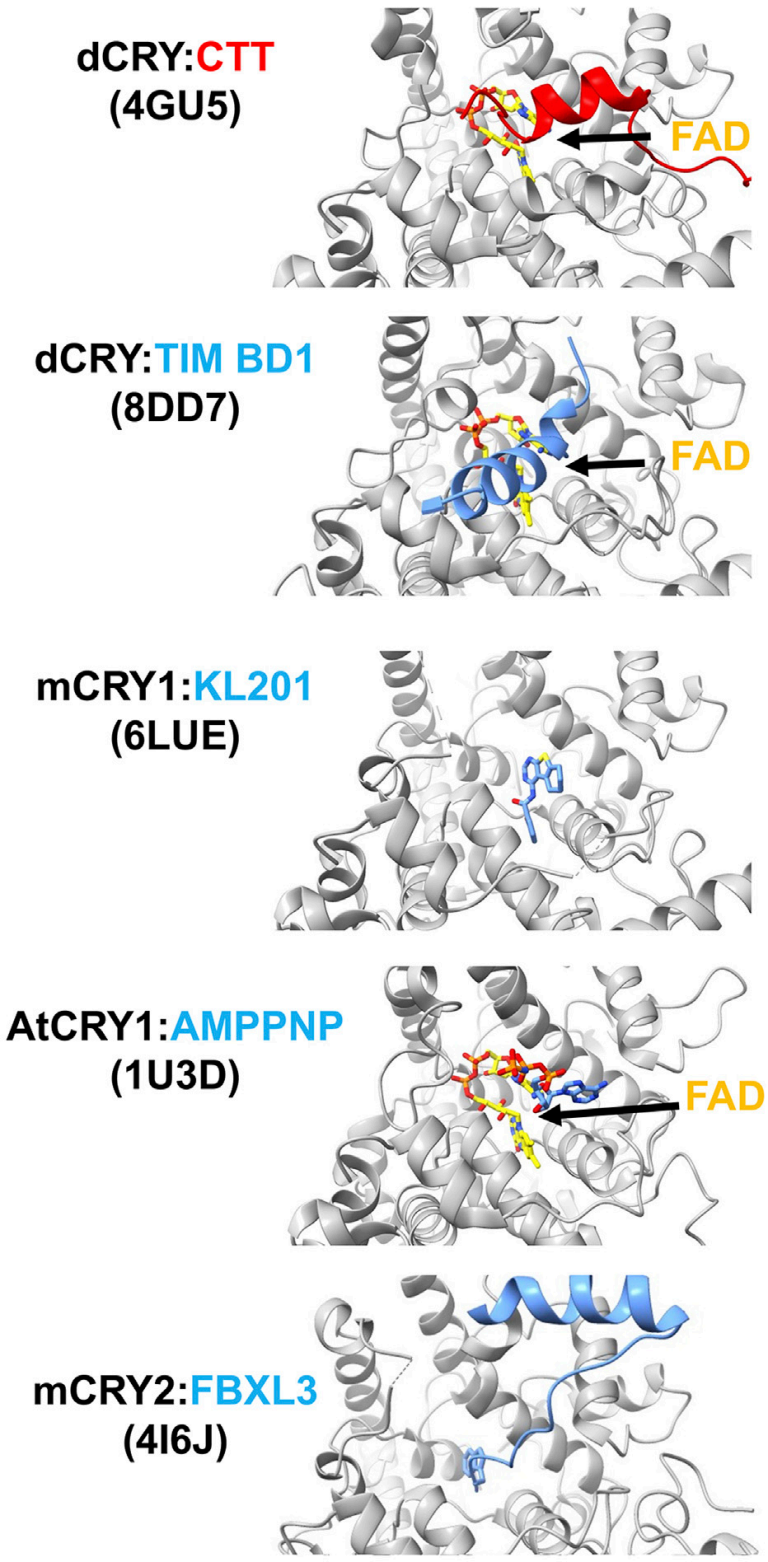
Interactions of cryptochromes in their primary pockets. When dCRY is in the dark state, its CTT rests in the FAD pocket. Upon light activation, the N-terminus of TIM binds dCRY by replacing the position of the dCRY CTT. The isoform-selective mCRY1 stabilizer, KL201, binds mCRY1 in the primary pocket. The non-hydrolyzable ATP analog AMPPNP binds in the plant CRY pocket. E3 ubiquitin ligase protein FBXL3 binds mCRY in the pocket to target degradation by the proteosome. FAD is shown in yellow and PDB codes of the structures are given in parentheses.

The CRY primary pocket binds the FAD cofactor (Lin, C. T., et al., 1995)), small molecule inhibitors (Nangle, S., et al., 2013) and protein partners (Khan, et al., 2012; Gao, Peng, et al., 2013; Xing, et al., 2013) (Figure 4). It largely forms at the interface of the capping helices (primarily α11) with the primase bundle and is perimetrically structured by the PBL, C-terminal lid and PM (Figure 3). In most CRYs (and PLs) this pocket hosts the FAD cofactor in a distinctive conformation, stacking the isoalloxazine ring system close to the adenine ring. In mCRYs, small molecule effectors that were initially discovered in screens for disruption of the transcriptional oscillator bind in the primary pocket (Hirota, et al., 2012; Nangle, S., et al., 2013). A large number of inhibitors have been developed that have selectivity for either of the two mCRY isoforms and exhibit considerable effects on circadian rhythms and other biological activity (Miller, et al., 2020a; Miller, et al., 2020b; Miller, et al., 2021). The small molecules have been shown to compete with E3 ubiquitin ligases, especially FBXL3, for the mCRY primary pocket. The ubiquitination of CRYs by FBXL3 (Busino, et al., 2007; Siepka, et al., 2007) and FBXL21 (Hirano, et al., 2013; Yoo, et al., 2013) leads to their degradation by proteasomes, providing a mechanism to reset the CLOCK:BMAL1 transcriptional cycle. In recognizing mCRY, FBXL3 inserts a C-terminal Trp residue directly into what would be the FAD-binding pocket (Xing, et al., 2013) (Figure 4). Small molecule inhibitors that stabilize mCRY *in vivo* block the site where the C-terminus of FBXL3 binds (Miller, et al., 2020a; Miller, et al., 2020b; Miller, et al., 2021) and correspondingly, knockdown of FBXL3 substantially limits the inhibitor effects (Miller, et al., 2020a). In addition to it’s C-terminus targeting the primary pocket, the leucine-rich repeats (LRRs) of FBXL3 encircle the CC-helix (Xing, et al., 2013), competing with interactions between mCRY and mPER2 (see below).

The primase bundle and C-terminal lid fashion both the primary and secondary pockets, which face roughly opposite directions. Switching these elements between mCRY1 and mCRY2 conveys the strong CLOCK:BMAL1-repressor characteristics of mCRY1 onto mCRY2 and visa versa (Khan, et al., 2012). Several specific residue substitutions at the base of these elements that impair repressor activity and reduce rhythm amplitude align more toward the face of the secondary pocket, but are distributed such that interfaces involved in CLOCK:BMAL1 interactions are likely to be extensive (Khan, et al., 2012).

In PLs, the extended pocket formed by the PBL, PM, and C-terminal lid accepts the substrate DNA lesion (Mees, et al., 2004; Glas, et al., 2009). As noted above, in dCRY the CTT binds next to the flavin in the primary pocket, and the CCEs of other CRYs may also interact here (Figure 4). Upon light activation the dCRY CTT is replaced by the N-terminus of the TIM circadian corepressor, which provides a remarkable structural mimic for the 6-4 lesion repaired by DNA photolyases (Lin, C. F., et al., 2023). In plant CRYs, ATP binds in this position and appears to stabilize the CCE against the PHR (Brautigam, et al., 2004) (Figure 4). Release of ATP upon light activation may be coupled to destabilization and release of the CCE (Cailliez, et al., 2014; Müller, et al., 2014; Eckel, et al., 2018; Iwata, et al., 2020). Notably, ATP and other metabolites also facilitate photoreduction of the plant CRY flavin, by either directly acting as reductants or making the protein more susceptible for photoreduction (Engelhard, et al., 2014). In mCRYs, substitutions of residues in and surrounding the PBL and C-terminal lid affect period length without affecting protein stability, i.e., E3 ligase interactions (Ode, et al., 2017).

The primary pocket is also a main conduit for oxygen to access the flavin cofactor (Mondal and Huix-Rotlant, 2019; Deviers, et al., 2024). Return to the dark-adapted state requires oxidation of the semiquinone, which can be mediated by reduction of molecular oxygen to superoxide and other reactive oxygen species (Müller and Ahmad, 2011; van Wilderen, et al., 2015; Arthaut, et al., 2017), that themselves may have important signaling roles (Jourdan, et al., 2015). Oxygen radicals have also been suggested as a paired spin with the flavin semiquinone in the radical pair mechanism of magnetic field sensing (Hogben, et al., 2009; Müller and Ahmad, 2011); however, stabilizing interactions within the pocket would be required to promote spin-relaxation times long enough for O_2_ to be a feasible player in magnetoreception (Hogben, et al., 2009; Player and Hore, 2019; Ramsay and Kattnig, 2022). Simulations find that superoxide localizes close to the flavin, by Trp A, flavin N5 and the N5-interacting residue (Asn391 in CRY IV), and on the other side of isoalloxazine ring by a conserved His residue (353) in the primary pocket (Deviers, et al., 2024). Behavioral data in birds (Wiltschko, et al., 2016) and plants (Pooam, et al., 2019; Hammad, et al., 2020) indicates that the magnetically sensitive reactions take place in the dark, following CRY illumination, and hence the implication of the oxidative reactions. There is also the possibility of O_2_ playing the role of a third spin in a modification of the radical pair mechanism, which again would require localization of O_2_ within the pocket (Ramsay and Kattnig, 2022). In dCRY, oxidation of the flavin is accompanied by some loss of FAD by the protein (Kutta, et al., 2018), which is unusual for flavoproteins, especially given the high affinity of dCRY for FAD (Foroutannejad, et al., 2023) and thus may have functional implications (Kutta, et al., 2018). With respect to the role of the primary pocket in magnetoreception, surprisingly, the 52 C-terminal residues of dCRY appear sufficient to mediate magnetic field responses in flies (Bradlaugh, et al., 2023). This region of the protein corresponds to largely just the foot helix and the short CCE, which is unlikely to maintain an ordered structure. It is currently unclear how such a mechanism would operate and it is not without controversy (Bassetto, et al., 2023).

In PLs, the secondary pocket, formed by the cleft between the nucleotide-binding site of the α/β domain and the α-helical domain accommodates the antenna cofactors (Figure 3). The variety of these moieties, which harvest light to accentuate excitation of the flavin for redox-mediate DNA repair, continues to grow and includes 5,10-methenyltetrahydofolate (MTHF), 7,8-didemethyl-8-hydroxy-5-deazariboflavin (8-HDF, also known as F0), flavin mononucleotide (FMN), FAD, and 6,7-dimethyl-8-ribityl-lumazin (DLZ) (Geisselbrecht, et al., 2012; Ozturk, N., 2017). CRYs do not generally bind antenna cofactors, except the CRY-DASH family, members of which maintain DNA repair capabilities (Bayram, et al., 2008; Pokorny, et al., 2008; Kiontke, Stephan, et al., 2020). Nonetheless, there were original reports of dCRY binding some MTHF (Berndt, et al., 2007), and it has been noted that plant CRYs conserve two MTHF-binding Trp residues found in class III PLs (related to plant CRYs) (Scheerer, et al., 2015). The secondary pocket mediates interactions between mCRY and its transcriptional target CLOCK (Michael, et al., 2017b; Rosensweig, et al., 2018; Fribourgh, et al., 2020). The secondary pocket has been called “an evolutionary hotspot”, wherein subtle residue changes alter the ability of Type II CRYs to repress CLOCK:BMAL1 and thereby affect circadian rhythms (Rosensweig, et al., 2018). In particular, minor residue variations in the serine loop at the rim of the secondary pocket affect CRY stability, nuclear import and CLOCK repression (Fribourgh, et al., 2020; Parlak, Gizem Cagla, et al., 2022). Type IV vertebrate CRYs cannot rescue the circadian clock function of Type II CRYs, perhaps because of residue changes that block access to the secondary pocket (Zoltowski, et al., 2019).

## Interaction modes of binding partners: beyond the pockets

**FIGURE 5:**
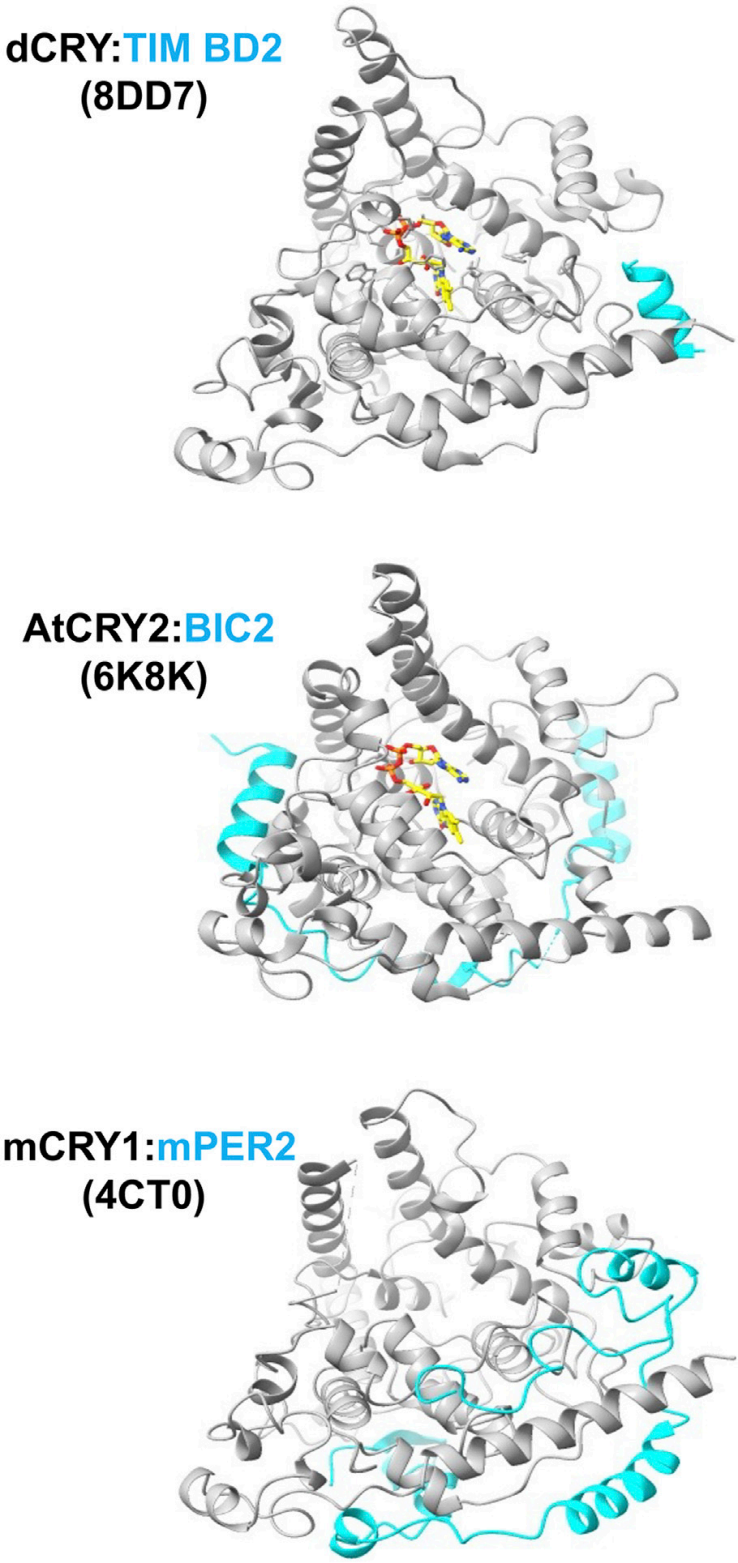
Interactions of cryptochromes at their peripheral sites. The C-terminus of TIM binds dCRY by the foot helix, similar to where regions of mPER bind mCRY. BIC2 wraps around the periphery of plant CRY. PDB codes of the structures are given in parentheses.

Key contacts of partners with CRYs involve peripheral regions of the PHR domain and not necessarily the primary and secondary pockets (Figure 5). mCRY1 and mCRY2, pivotal components of the circadian clock negative feedback loop, exert their regulatory influence by inhibiting the transcriptional activity of the CLOCK:BMAL1 complex (Khan, et al., 2012; Michael, et al., 2017a). mCRY1 interacts directly with the CLOCK:BMAL1 PAS-AB core and multivalent interactions between CLOCK PAS-B and the BMAL1 TAD are also required for mCRY1-mediated repression (Xu, Haiyan, et al., 2015; Michael, et al., 2017b).

mCRYs also associate with the mPER proteins to form the central repressor complex (Takahashi, 2017). The mCRY:PER interaction has been characterized crystallographically for the C-terminal regions of mPER2 with mCRY1 (Schmalen, et al., 2014; Michael, et al., 2017a) and mCRY2 (Nangle, S. N., et al., 2014). The mPER2 binding region forms extended polypeptide segments that wrap the protein behind the flavin pocket to the clasp region and particularly engage the C-terminal CC helix in a coiled-coil-like interaction (Figure 5) that is consistent with prior mutagenesis studies probing the mCRY2:PER2 contact (Ozber, et al., 2010). In one structure, a C-terminal β-hairpin of the mPER2 binds alongside of the serine loop of mCRY1 (Schmalen, et al., 2014). The interaction between mCRY1 and mPER2 is further mediated by a zinc ion that tetrahedrally coordinates Cys414 from the C-terminal lid with His473, and two Cys residues on mPER2 (residues 1210 and 1213) (Nangle, S. N., et al., 2014; Schmalen, et al., 2014). This intermolecular zinc site has been designated a “zinc-finger” (Nangle, S. N., et al., 2014), although it has tertiary structure and ligand spacing that differ from nucleic-acid binding CCCH-type zinc fingers. A neighboring mCRY1 disulfide bond involving Cys363 and Cys412 that forms in the unbound structure, breaks to potentially allow the conformational changes and dynamics required for the zinc site to form, thereby providing the means for redox state to mediate mPER binding (Schmalen, et al., 2014). The zinc site contributes to the mCRY:mPER binding affinity *in vivo* (Schmalen, et al., 2014), but substitutions of the ligating residues only have minimal effects on molecular rhythms (Nangle, S. N., et al., 2014). Both mCRY2 and PER2 cysteine residues are sensitive to oxidative conditions that could alter their interactions via the disulfide/zinc ion switch (Baidanoff, et al., 2022), but the full functional impact of such a mechanism remains to be explored. Alternations to mCRY upon mPER binding are particularly evident in the C-terminal lid, the serine loop, and other C-terminal structural elements. Notably, a naturally occurring human CRY2 (hCRY2) variant (Ser420Phe) at the junction of the primase bundle and C-terminal lid curtails repressor activity by reducing hPER2 affinity, but also reduces nuclear import of hCRY2 (Parlak, G. C., et al., 2023).

dCRY also participates in interactions that involve regions distinct from the primary and secondary pockets. For example, in addition to its principle interaction mode with the primary pocket, the *Drosophila* TIM protein supplies a peripheral helix to bind dCRY in regions similar to where the mPERs bind mCRY (Lin, C. F., et al., 2023). In a novel form of association, some evidence suggests that the iron-sulfur-cluster-containing protein designated MagR complexes with dCRY to form a rod-shaped, magnetically sensitive protein complex; however, the resolution of the structural characterization was insufficient to fully define this interaction (Qin, et al., 2016). dCRY also influences neuronal firing in a relatively fast response that involves its functional coupling to the voltage-gated potassium channel β-subunit (Kvβ), known as hyperkinetic (HK) (Baik, et al., 2019; Fogle, K. J., et al., 2015). Molecular dynamics-guided modeling of the dCRY:HK complex associates Trp D of the dCRY Trp tetrad in the α-helical domain close to the presumed binding site of NADPH in HK, thereby providing a mechanism for direct coupling between dCRY photochemistry and redox regulation of HK channel activity (Hong, et al., 2018).

In plants, interactions of the Blue-light Inhibitor of Cryptochromes (BIC) proteins block light activation by also engaging in peripheral interactions outside of the primary and secondary plant CRY pockets (Wang, et al., 2017a; Wang, et al., 2018b; Ma, et al., 2020b) (Figure 4). BICs specifically engage the PHR subdomains, adopting a 'U-shaped lock' conformation that impedes oligomerization and interactions with signaling partners (Ma, et al., 2020a). The BIC interaction involves PHR regions quite different from those of the PER proteins, with key contacts on the opposite side of the PHR (Ma, et al., 2020b). For example, BIC2 interacts with the C-terminal clasp region of plant CRY2 in a manner that prevents this domain from mediating tetramerization (Ma, et al., 2020a).

## Type I cryptochrome light activation: flavin reduction and CTT undocking

**FIGURE 6:**
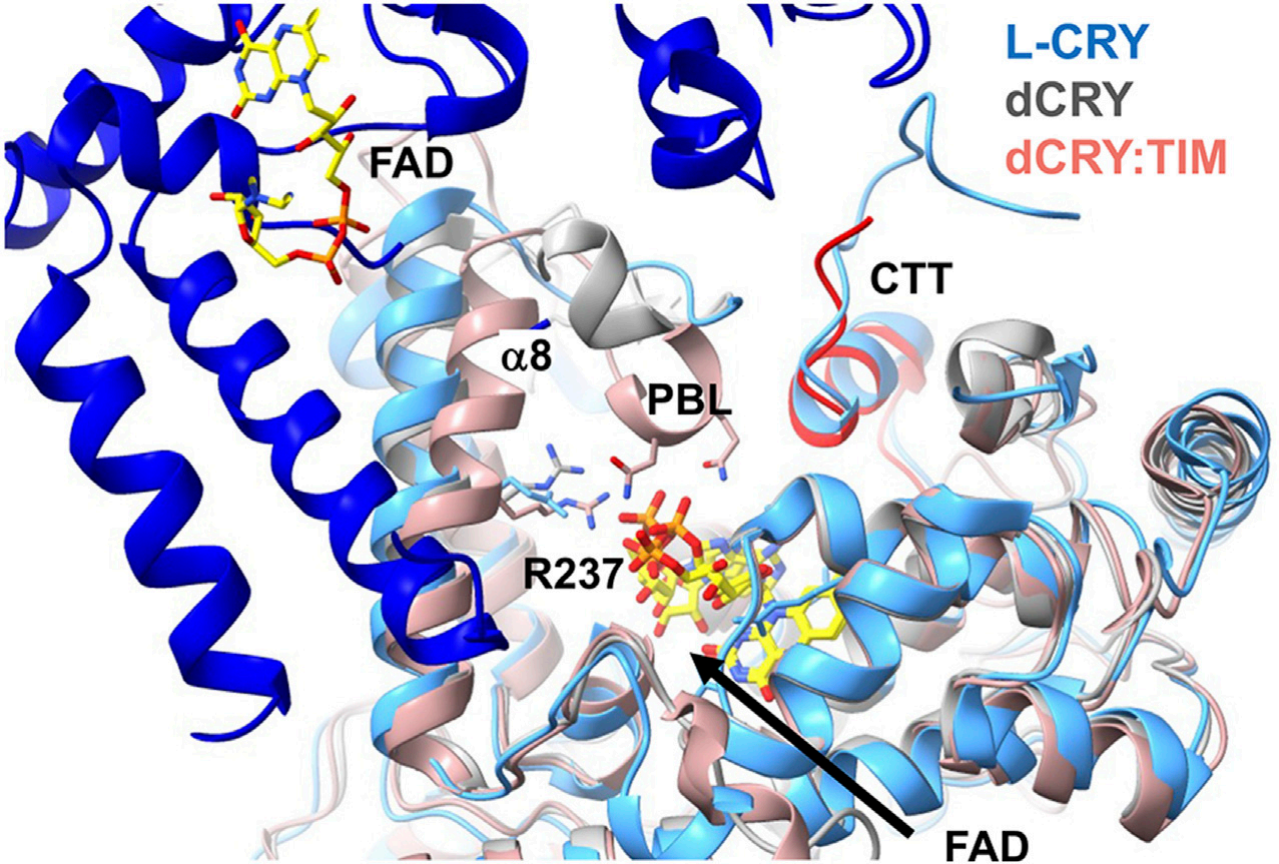
Conformational changes in Type I CRYs. Superposition of the structures of dark-state dCRY (grey) and light-state dCRY bound to TIM (red) show that Arg238 and α8 shift toward the rearranged phosphate groups of FAD. The PBL also collapses into the pocket. Also superimposed is the dark-state dimer of L-CRY. L-CRY conserves the analog of Arg238 and its movement toward the flavin may disrupt the dimer contact formed by α8. The longer CCE of L-CRY also contributes to the dimer interface. The CCE of L-CRY contains a helix that aligns with the CTT of dCRY (red) that displaces upon light activation.

The mechanisms by which light activation propagates conformational signals in cryptochromes for their signaling functions has been most extensively studied in Type I CRYs and plant CRYs because these systems have both been structurally accessible while also having clearly defined signaling functions in their respective organisms. As noted, there is little evidence that the vertebrate Type II CRYs are light sensors (Kutta, et al., 2017), although they may interact with flavin in modes that allow for responses to redox and metabolic state (Hirano, et al., 2017; Calloni and Vabulas, 2023). In the case of Type 1 invertebrate CRYs, blue-light absorption by the oxidized flavin produces the anionic semiquinone by photoreduction via the Trp triad/tetrad (Berndt, et al., 2007; Kao, et al., 2008; Kutta, et al., 2018; Ozturk, N., et al., 2014; Ozturk, N., Selby, C.P., Annayev, Y., Zhong, D., Sancar, A., 2011; Song, et al., 2007; Vaidya, et al., 2013). dCRY mediates the degradation of the transcriptional co-repressor TIM (Koh, et al., 2006; Lin, F. J., et al., 2001) and its own light-driven self-degradation (Busza, et al., 2004; Dissel, et al., 2004; Hemsley, et al., 2007). These processes require light-gated interactions with E3 ubiquitin ligases, of which two have been implicated: Jetlag (Peschel, et al., 2009) and Ramshackle (Brwd3) (Ozturk, N., et al., 2013). For dCRY, variants in the Trp triad with varying light sensitivities correspondingly affect the ability to degrade TIM and undergo self-degradation (Lin, 2019). The CCE of dCRY gates interactions with targets. dCRY has one of the shortest CCEs, and the structure of the full-length protein revealed that the majority of this element forms a C-terminal tail helix that inserts into the flavin pocket (Zoltowski, et al., 2011b; Czarna, et al., 2013; Levy, et al., 2013). Proteolytic sensitivity measurements identified regions in the CTT and surrounding CTT-coupled motif (i.e., the C-terminal Lid, PM and PBL) that become differentially sensitive upon light activation (Vaidya, et al., 2013). Spin-labeling studies in concert with pulse-electron-spin resonance spectroscopy measurements demonstrate that undocking of the CTT depends on forming the FAD anionic semiquinone (ASQ), and this undocking was recapitulated in molecular dynamics (MD) simulations (Berntsson, et al., 2019; Chandrasekaran, et al., 2021; Ganguly, et al., 2016; Lin, C., et al., 2022). Time-resolved SAXS studies of dCRY were also consistent with CTT displacement upon ASQ formation (Berntsson, et al., 2019). The precise mechanism of CTT undocking is not fully determined. Two conserved histidine residues that undergo protonation changes during the enzymatic reactions of photolyases may similarly respond to flavin photoreduction (Ganguly, et al., 2016). These residues have been shown to affect the fidelity of CRY interactions with its target TIM, but their substitution does not block CTT release (Berntsson, et al., 2019; Lin, C., et al., 2022). The cryo-EM structure of light-exposed dCRY bound to TIM defined key interactions of the signaling state (Lin, C. F., et al., 2023). The ∼1400 residue TIM proteins inserts its N-terminal helix into the flavin binding pocket, effectively replacing the CTT. The regions that change proteolytic accessibility (e.g., the PBL, PM, C-terminal lid and CTT) (Vaidya, et al., 2013) rearrange conformation upon TIM binding. TIM is a large helical protein made from armadillo (ARM) repeat modules, wherein the first 3 ARM repeats make extensive interactions with dCRY. Moreover, a C-terminal helix of TIM that resides in an otherwise disordered region binds to a separate dCRY element near the C-terminal foot (CC) helix. The dCRY FAD substantially rearranges in the TIM complex, particularly the diphosphate groups which change orientation to interact with an Arg residue (258) on helix α8, which inserts further into the active site to replace the otherwise coordinating Mg^2+^ ion (Figure 6). The PBL refolds from the surface into the flavin pocket, with its TIM-bound conformation stabilized by His377, one of the two conserved His residues that influences TIM recognition and tail release (Figure 6). Peptide binding assays with the N-terminal region of TIM show that Arg237 and the residues in the PBL that undergo the most change are critical not only for TIM binding, but also for CTT undocking, suggesting that the ASQ state of the flavin promotes the movement of Arg237 and the PBL prior to CTT displacement (Schneps, et al., 2024). Indeed, the TIM-bound conformation of the PBL clashes with the CTT when it is bound in the flavin binding pocket (Schneps, et al., 2024). MD simulations of dCRY activation in the absence of TIM suggest that a key salt bridge involving a highly conserved Arg-Asp pair that buttresses the isoalloxazine ring of the flavin disrupts when the flavin is photoreduced (Wang, et al., 2021a), consistent with the cryoEM structure (Lin, C. F., et al., 2023). Similar movements in this salt-bridge have been observed in serial crystallography experiments of photoreduced PLs (Maestre-Reyna, et al., 2022; Cellini, et al., 2024). Finally, the dCRY PHR domain alone binds the N-terminal peptide, independent of light activation, but the affinity for the peptide increases in the light (Schneps, et al., 2024). Thus, light-dependent CTT release is not the only factor involved in the increase of CRY affinity for TIM in the photoreduced state.

Type IV avian CRY displays some intriguing similarities and differences with respect to their conformational activation mechanism compared to dCRY. Pigeon CRY4 (ClCRY4) binds FAD, forms a neutral semiquinone when photoreduced and has a similar length CCE compared to dCRY (Xu, J. J., et al., 2021; Zoltowski, et al., 2019). Proteolytic protection assays guided by structural analysis indicated that the PBL becomes more protected in the semiquinone form (Zoltowski, et al., 2019). A closed PBL or “gate” in the semiquinone state is also consistent with all-atom (Schuhmann, et al., 2021) and course-grain MD simulations of ClCRY4. This movement of the PBL in the MD simulations produced a state similar to the collapsed PBL conformation of dCRY observed in the TIM complex. However, the CCE of ClCRY behaviors opposite to the CCE of dCRY upon photoreduction, with the former also becoming more protected in the flavin photoreduced state (Zoltowski, et al., 2019; Schuhmann, et al., 2024), whereas the latter undocks to reveal the TIM binding pocket (Chandrasekaran, et al., 2021).

A variant Type I CRY (L-CRY) found in the marine bristle worm *Platynereis dumerilii* paces reproduction to the lunar cycle (Poehn, et al., 2022; Zurl, et al., 2022). L-CRY displays an intriguing modification to the dCRY activation mechanism (Vu, et al., 2023). In this case, the dimeric L-CRY associates in the dark-state through an interface that involves α8 and interactions between the CCE and the clasp region (Figure 6). The protein is proposed to undergo a two-step light activation process. At low light levels only one subunit photoreduces, sending a specific signal within the nucleus (Poehn, et al., 2022). Upon photoreduction of both subunits at higher light intensity and reduced quantum yield, the protein dissociates into subunits, revealing a nuclear export signal. Asymmetric activation at low light and high quantum yield is proposed to produce a signaling state that then distinguishes dim moonlight from higher intensity sunlight. The addition of a terminal Tyr residue to the Trp tetrad may stabilize charge-separated radicals to a greater degree and fine-tune light sensitivity (Vu, et al., 2023). Indeed, manipulation of the Trp -tetrad in dCRY can also increase light-sensitivity (Lin, C. F., et al., 2018). Based on the structure of the dimeric dark state, the dimer interface may be disrupted by CTT undocking and a shift of α8 when conserved Arg (dCRY 237, L-CRY 234) responds to flavin photoreduction analogous to that observed in dCRY (Schneps, et al., 2024) (Figure 6). Notably, family members of other flavoprotein light-sensors such as LOV domains also show either light-dependent associations or dissociations by coupling similar flavin pocket chemistry to perturbations of peripheral structural elements that increasingly diverge from the cofactor pocket to the protein interaction surface (Vaidya, et al., 2011; Conrad, et al., 2013; Conrad, et al., 2014).

Animal-like CRYs (aCRYs) from algae also undergo light-depend rearrangements of their CCEs. Although the sequences of CCEs are very different from Type I CRYs they also appear to associate with the flavin pocket in the dark and become displaced in the light. aCRY accesses the oxidized, semiquinone and hydroquinone flavin states which can be interconverted by blue and red light, respectively (Franz, et al., 2018; Kottke, et al., 2017; Li, P., et al., 2022; Spexard, et al., 2014). HDX studies indicate a modest change in PHR protection upon conversion to the fully-reduced hydroquinone state due to diminished interactions of the CCE with the PHR (Franz-Badur, et al., 2019). However, Fourier transform infrared (FTIR) spectroscopy did not reveal substantial conformational changes between either the FADox and FADH° states or the FADH° and FADH^—^states (Spexard, et al., 2014; Oldemeyer, et al., 2016). On the other hand, FRET studies indicate a CCE displacement by ∼ 15 Å upon blue-light irradiation to the presumed neutral semiquinone (Li, P., et al., 2022).

## Plant cryptochrome light activation: CCE undocking and oligomerization

Light activation of plant CRYs has been informed by recent cryoEM and crystal structures of the protein in dark and light-exposed states (Hao, et al., 2023; Liu, B. B., et al., 2016; Ma, et al., 2020a; Ma, et al., 2020b; Palayam, et al., 2021; Shao, et al., 2020; Wang and Lin, 2020a). The first cryptochrome crystal structure was of the AtCRY1 PHR domain (1U3C) (Brautigam, et al., 2004); it displays the characteristic photolyase fold as well as a head-to-head (HH) association in the crystal involving the α/β domain with α10-α11 that has been found to manifest in nearly all of the other structures determined by crystallography or cryoEM (see below). Light photoreduces AtCRY1 and AtCRY2 to primarily the neutral or protonated FAD semiquinone state via a Trp triad (Giovani, et al., 2003; Zeugner, et al., 2005; Kottke, et al., 2006; Banerjee, et al., 2007). Although there has been debate on the identity of the key light-activated state within the cell (Li, X., et al., 2011), there are strong arguments that the NSQ is the functionally relevant light-adapted state (see (Ahmad, 2016) for a thorough discussion of this issue). Furthermore, NSQ formation clearly promotes both changes in interactions between the CCE and the PHR (Goett-Zink, et al., 2021; Kondoh, et al., 2011; Partch, C. L., et al., 2005) as well as the oligomerization of both full-length CRY proteins and their PHR domains *in vitro* (Ma, et al., 2020a; Palayam, et al., 2021). Plant CRY PHRs form both dimers and tetramers, with the tetrameric state favored by the photoreduced flavin as well as other conditions (Shao, et al., 2020). These tetramers are also preferentially formed by variant proteins with single residue substitutions (W368A AtCRY2 or W374A in *Zea mays* (ZmCRY1)) that result in light-independent activation *in vivo* (Shao, et al., 2020). Notably, AtCRY1 has also been found to oligomerize independent of light through chemical cross-linking studies in plant seedlings (Sang, et al., 2005); however, more recent quantitative pull-down experiments show that both AtCRY1 and AtCRY2 oligomerization is enhanced by light *in vivo* (Liu, Q., et al., 2020; Wang, Q., et al., 2016). Diffusion measurements suggest that AtCRY1 does not oligomerize with light, but rather undergoes a large conformational change involving the CCE (Kondoh, et al., 2011), although these measurements are sensitive to both conformation and oligomeric state.

**FIGURE 7:**
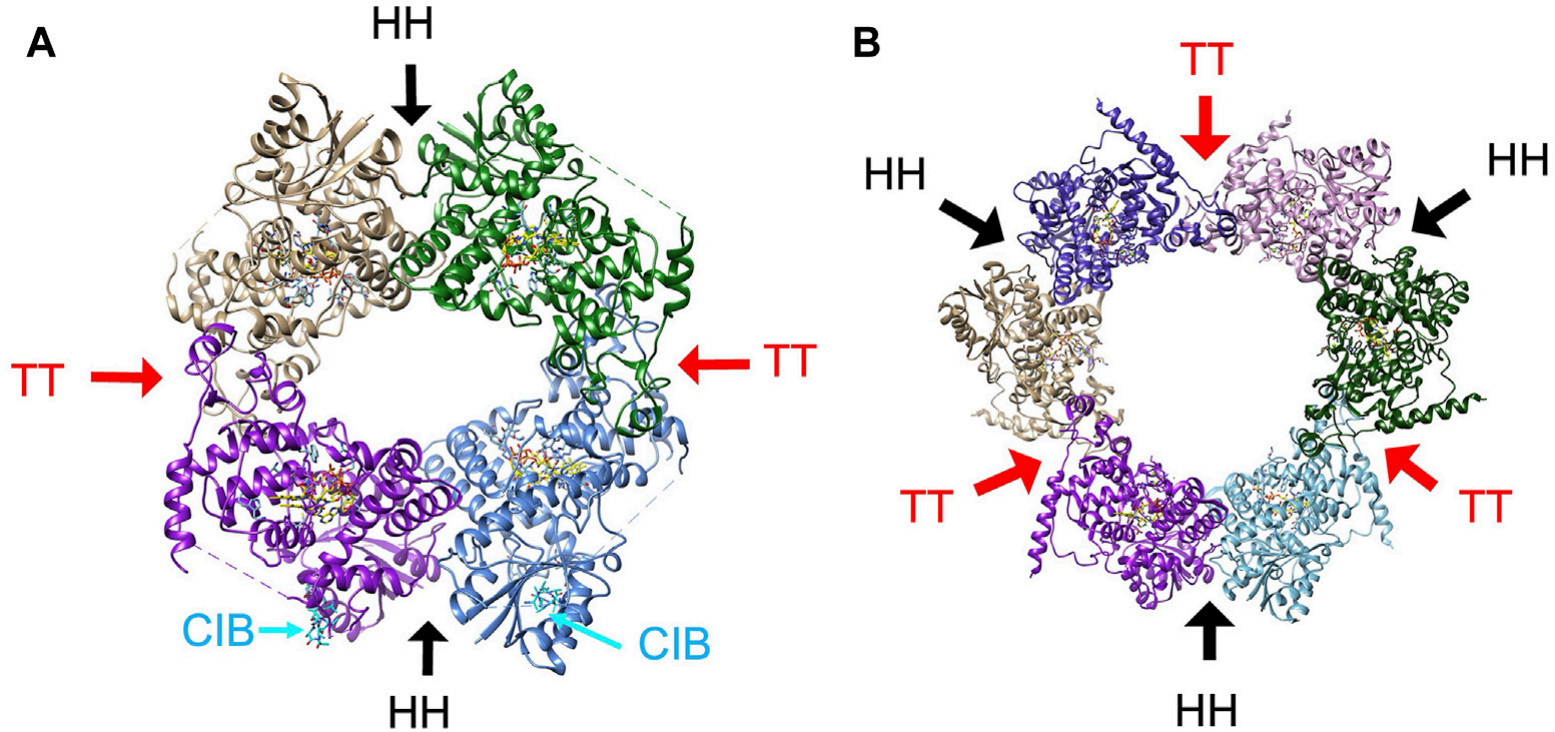
The oligomeric states of plant CRY. **(A)** The tetramer characteristic of activated plant CRY1 and CRY2 has two subunit interfaces, one involving the α/β domain, connector and Cap (HH, black arrows) and the other involving the Clasp region (TT, red arrows). Cryo-EM electron density indicative of CIB1 helix binding (cyan) to AtCRY2 was found near the HH interface (PDB:7x0Y). **(B)** The hexamer that composes the crystal lattice of AtCRY1 PHR domain (PDB;1u3c) maintains the HH interface and a similar TT interface as found in the tetrameric structures of full-length plant CRY1 and CRY2.

The tetrameric state of plant CRY, which has been observed for both CRY1 and CRY2 of several plant species (Figure 7), are composed of two distinct interfaces (Ma, et al., 2020b; Shao, et al., 2020; Palayam, et al., 2021). The plant CRY tetramer has 222 symmetry, which produces a head-to-head (HH) contact and a tail-to-tail contact (TT) (Figure 7). The TT interface has been called a head-to-tail contact (HT), but such a description is usually reserved for oligomers in which the twofold symmetry axis does not reside within the interface itself and hence pairs one region of the protein with another, which is not the case for the AtCRY tetramer. Furthermore, the two interfaces have been interchangeably described as interface one and interface 2. Here we will refer to HH and TT interfaces which respectively correspond to the HH and HT of Palayam et al. (Palayam, et al., 2021), interface two and interface one of CRY1 in Shao et al. (Shao, et al., 2020)) and interface one and interface two of Ma et al. (Ma, et al., 2020a). The HH interface primarily involves the α/β domain and associated connector region preceding the α5-α6 finger motif and the α10 capping helix of the α-helical domain (Figure 7). The TT interface is formed by a symmetric interaction of the irregular clasp helices (α18 and α19) that extend from the C-terminal lid and includes contacts from a region of the connector that precedes α8 and the C-terminus of α13 (Figure 7).

There is good evidence that light affects association about the TT interface. Single residue substitutions in this region (AtCRY R439L, W349A on α13) prevent light-induced tetramerization of AtCRY2, reduce interactions with a CIB1-derived peptide and only partially rescue the long hypocotyl phenotype by AtCRY2 in a *cry1* mutant (Shao, et al., 2020). In addition, genetically selected mutants that map to the TT interface of AtCRY1 (G347R, A462V) and CRY2 (G254R, P339L) disrupt light-independent oligomerization *in vivo* (Liu, Q., et al., 2020; Sang, et al., 2005) and Q333A and E462A at the TT interface reduce the formation of photobodies (Ma, et al., 2020a). Mutations at both the HH and TT interfaces reduce AtCRY2 photo-oligomerization *in vitro* (Ma, et al., 2020b). As noted above, BIC2 wraps the PHR domain as an extended polypeptide of disconnected helices to partially block the TT association (Ma, et al., 2020a). BIC2 binds to AtCRY2 in the dark and inhibits flavin photoreduction by perhaps slightly altering the position of the Trp-triad and flavin-protonating Asp residue (Ma, et al., 2020b). When illuminated by blue light, AtCRY2 targets the transcriptional regulator CIB1 (cryptochrome-interacting basic helix-loop-helix 1) through the PHR domain to regulate flowering (Liu, H. T., et al., 2008). CIB1 appears to bind near the HH interface (Figure 7), although the detailed structure of the protein could not be discerned in cryo-EM maps, which only revealed modest density for a helix-like structure (Hao, et al., 2023). The HH interface forms in the absence of light and thus may be available to bind CIB1 in the dark (Shao, et al., 2020). However, light-dependent TT oligomerization would recruit four CIB1 proteins together, which may be required for their transcriptional activation activity.

Of considerable interest are the conformational changes that propagate from the flavin to transform the plant CRY structure into the signaling state. Spectroscopic studies have determined the primary reactions of light activation in plant CRYs (reviewed in (Goett-Zink, et al., 2021; Aguida, et al., 2024)). In brief, flavin photoreduction to the ASQ precedes flavin protonation from a conserved Asp residue (Thoing, et al., 2013; Hense, et al., 2015; Thöing, et al., 2015), which ionizes in the dark-to-light transition. Infrared difference spectroscopy identified secondary structural regions affected by the electron and proton transfers at the AtCRY1 active center; early structural intermediates involve helical and turn rearrangements followed by later stage remodeling of the β-sheet within the α/β domain (Goett-Zink, et al., 2021). Like Type I CRYs, light releases the CCE from the PHR to gate downstream effects (Partch, C. L., et al., 2005; Sang, et al., 2005; Wang, et al., 2017b), and the spectral perturbations in the β-sheet likely reflect loss of interaction with the CCE. Phosphorylation or modification of the CCE also diminishes its interaction with the PHR domain, modulates its photosensitivity and activates downstream signaling (Yu, et al., 2007; Yu, et al., 2009; Wang, et al., 2017a). Notably the light-dependent TT interface is far removed from the α/β domain where spectroscopic changes reside, but the CCEs are large. One then might expect that CCE release impacts association about the TT interface. However, the PHR domains alone undergo light-activated tetramerization (Ma, et al., 2020a; Palayam, et al., 2021) without the need for CCE gating. In all, it is not yet entirely clear how flavin photoreduction generates a functional signaling state. Under physiological conditions, tetramerization may be a necessary, but insufficient condition to activate plant CRY. Flavin photoreduction could regulate both CCE release and other structural changes that encourage association about the TT interface. Oligomerization may also be important for the CCE itself to send signals because artificially induced CCE dimerization in the absence of the PHR alters gene expression in plants (Rosenfeldt, et al., 2008).

To better understand the molecular mechanism of plant CRY light activation, light-state crystal structures (Palayam, et al., 2021), cryoEM structures of light-activated CRY2 alone (Ma, et al., 2020b) and with CIB1 (Hao, et al., 2023), as well as cryoEM structures of activating mutants (Shao, et al., 2020) have been compared to dark-state structures, primarily determined in crystals (6K8I, 1U3C) with and without BIC2 (Brautigam, et al., 2004; Ma, et al., 2020a). Comparisons between the CRY2 light-state tetramer to the CRY2 crystal structure in darkness (PDB: 6K8I) identified several residues that appear to change position slightly between the clasp and the flavin pocket (Ma, et al., 2020b). These residues were then substituted, most with little effect; however, two substitutions Y232A and W353A promoted tetramer formation in the dark. The challenge with these analyses is that the dark state structures typically form the same or similar oligomeric states in crystals as the light state structures. Hence, crystallization may favor conformations and subunit assemblies found in the light-state. For example, 6K8I, although referred to as a monomer structure, forms a similar tetramer in the crystal lattice as the light-activated structure (Ma, et al., 2020a; Ma, et al., 2020b). This recapitulation of light-state-like oligomers in crystals is also the case for the original dark state of CRY1 PHR, which does not form a tetramer in the crystal, but does form a hexamer with very similar HH and TT interfaces (Brautigam, et al., 2004) (Figure 7). The lower resolution of the cryoEM structures also makes it challenging to compare more subtle conformational changes in the dark and light state. If one superimposes the individual subunits of the various structures there does not appear to be a consistent pattern that distinguishes light (or mutationally activated) subunit conformations from the dark-state ones. The lower resolutions of the cryo-EM structures also limits evaluation of changes in flavin conformation.

The Trp triad may also participate in propagating conformational changes from the initial photochemistry. The activating mutations of W374A (CRY1) and W368A (CRY2) involve one of the Trp-triad residues. Photoactivation is blocked in these variants, but conformational changes induced by the residue substitutions appear to activate oligomerization (Engelhard, et al., 2014). The structural perturbations are removed from the HH and TT interfaces, but they do interact with the connector region and thereby could affect the clasp. Whether a chemical change in the Trp triad (e.g., a cation or neutral radical) is stable long enough to drive a change in oligomerization that must act on the timescale of physiological signaling is an open question. Going forward, the structure of a dark-state plant CRY monomer with an intact CCE would provide an important reference for further understanding plant CRY photoactivation.

## Summary and Outlook

Despite their diversity of function, CRYs share a remarkably conserved structure with two domains whose general folds are largely invariant. This architecture provides two pockets to accommodate cofactors, small molecules and partner proteins. The CRY conformation, particularly involving the CCE and the PBL, responds to moieties that bind in the pockets, thereby gating pocket access or revealing interaction motifs at the periphery or on the CCE itself. Photoreduction of FAD bound in the primary pocket to a semiquinone state (anionic or neutral) alters the protein properties in complex ways that share some commonalities among CRYs close to the cofactor but then diverge as more peripheral structural elements differ. Photoreduction utilizes an internal chain of Trp and Tyr residues that enables stabilization of charge separation over a relatively long distance and time. It remains to be fully understood how changes in flavin and Trp redox states propagate to other regions of the protein such as the CCE, clasp and α/β domain to regulate new interactions or facilitate changes in oligomeric state, although considerable details are becoming apparent. Non-photoresponsive CRYs, such as the mammalian Type II proteins, couple pocket reactivity to conformation in distinct ways whose details continue to be revealed, whereas the CRY-DASH family couple signal transduction with DNA repair activities. Thus, the understanding of the structure and mechanism of CRYs informs on many biological processes. This review has not discussed the application of cryptochromes to bioengineering applications, such as in the area of optogenetics (de Mena, et al., 2018; Lu, et al., 2020; Yamada, et al., 2020; Iwata and Masuda, 2021; Aguida, et al., 2024), but as new mechanisms reveal themselves, there is increasing potential to utilize cryptochromes as versatile sensors and control elements in entirely new processes that may reach beyond biology and into the realms of material science, device design and information processing.
